# Effect of topical nitric oxide donors 0.03% nitroglycerin and 0.1% hydralazine on intraocular pressure in healthy canine eyes

**DOI:** 10.1002/vms3.945

**Published:** 2022-09-22

**Authors:** Parker A. Wilcox, Travis D. Strong, Lionel Sebbag, Rachel A. Allbaugh

**Affiliations:** ^1^ Department of Veterinary Clinical Sciences, College of Veterinary Medicine Iowa State University Ames Iowa; ^2^ Present address: 1900 Coffey Rd Columbus OH 43210; ^3^ Present address: 510 Harry Walker Parkway South, Newmarket, Ontario, L3Y 0B3; ^4^ Present address: Koret School of Veterinary Medicine Hebrew University of Jerusalem PO Box 12 Rehovot 7610001 Israel

**Keywords:** canine, glaucoma, intraocular pressure, nitric oxide, nitric oxide donors

## Abstract

**Objective:**

To investigate the potential intraocular pressure (IOP)‐lowering effects of nitric oxide (NO)‐donating compounds in healthy canine eyes

**Methods:**

A total of 79 dogs were divided into 3 groups in a masked, controlled and randomised study. Group N (*n* = 26) was administered 0.03% nitroglycerin in one eye and vehicle‐control in the other, Group H (*n* = 26) was administered 0.1% hydralazine in one eye and vehicle‐control in the other, while Group C (*n* = 27) received vehicle‐control in both eyes (control group). Following eye drop administration, IOP was measured in both eyes at selected times (10–250 min), along with monitoring of heart rate and signs of ocular discomfort. Data was analysed with repeated measures mixed model and one‐way ANOVA

**Results:**

IOP was significantly reduced over the 4‐h period with 0.03% nitroglycerin (*p* < 0.0001) but not 0.1% hydralazine (*p* = 0.520) when compared to contralateral vehicle‐controlled eyes. IOP was reduced by up to 12% with 0.03% nitroglycerin from 10 to 70 min post‐treatment; however, differences in IOP at individual time points were not statistically significant for either drug (*p* ≥ 0.133) as compared to contralateral vehicle‐control eyes. No treatment group significantly affected heart rate (compared to Group C), and both treatment groups appeared well tolerated

**Conclusions:**

Both compounds were well‐tolerated in healthy dogs. Nitroglycerin mildly reduced IOP in canine eyes, and further investigations are warranted in healthy and diseased states (e.g. glaucoma, ocular hypertension).

## Graphical abstract

Topical nitric‐oxide donors 0.03% nitroglycerin and 0.1% hydralazine were investigated for the potential effect on intraocular pressure in normal canine eyes. This study determined healthy canine eyes that received 0.03% nitroglycerin had a significantly lower IOP over the 4‐hour testing period when compared to vehicle‐control eyes, although the maximal IOP reduction was somewhat limited (12%). No significant reduction in IOP was identified at any time point in eyes that received 0.1% hydralazine.

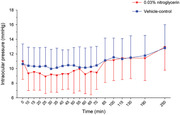

## 1 INTRODUCTION

Canine glaucoma is a common, often painful, and potentially blinding ophthalmic condition that is generally associated with elevated intraocular pressure (IOP). Glaucoma leads to retinal ganglion cell degeneration, damage to the optic nerve and eventually irreversible blindness (Gelatt et al., 2014; Komaromy et al., 2019). The goals of medical therapy are to maintain IOP in a normal range and prevent intraocular damage, but medical therapy often fails over time, leading to elevated IOP and damage to intraocular structures (Komaromy et al., 2019). Topical glaucoma therapeutics mainly used in canine ophthalmology include prostaglandin analogues, carbonic anhydrase inhibitors, and beta‐blockers (Gelatt et al., 2014). Additional drug classes that have been investigated include alpha2‐adrenergic agonists, direct and indirect acting parasympathomimetic agents, calcium channel blockers, and hyperosmotic agents (Gelatt et al., 2014). Current commonly used anti‐glaucoma therapies for this disease target one of two mechanisms of IOP regulation: aqueous humor (AH) production or the uveoscleral pathway of AH outflow (Cavet et al., 2014). Few treatments exist that preferentially influence the conventional pathway of AH outflow, which accounts for up to 85% of AH removal from the normal canine eye (Cavet et al., 2014; Ellis et al., 2009; Miller, 2008). Drug classes also known to largely target the conventional pathway of AH outflow and increase trabecular meshwork outflow facility include Rho Kinase inhibitors and muscarinic agonists (Gelatt et al., 2014; Komaromy et al., 2019). Relevant to this study, nitric oxide (NO)‐donating compounds have also been investigated and proven to influence the conventional outflow pathway (Komaromy et al., 2019).

Preliminary work in ex vivo, in vivo and laboratory animal studies have demonstrated the effect of NO on AH dynamics and conventional outflow physiology (Behar Cohen et al., 1996; Chang et al., 2015; Dismuke et al., 2008; Gabelt et al., 2011; Heyne et al., 2013; Nathanson, 1988; Nathanson, 1992; Osborne et al., 1993; Pianka et al., 2000; Rogers & Isenberg, 2015; Schneemann et al., 2002; Schneemann et al., 2003; Wiederholt et al., 1994). In live animals, multiple studies have identified a reduction in IOP after application of topical NO donors in rabbits, rats and non‐human primates (Behar Cohen et al., 1996; Borghi et al., 2010; Kotikoski et al., 2002; Muenster et al., 2017; Nathanson, 1988; Wang & Podos, 1995). In beagles with primary open angle glaucoma, several studies showed NO‐donating compounds (e.g. latanoprostene bunod) to be efficacious in lowering IOP (Borghi et al., 2010; Krauss et al., 2011). An abstract presented by Burn et al. (2020) described the effectiveness of latanoprostene bunod in significantly reducing IOP and increasing outflow facility in normal and ADAMTS10 open angle glaucoma dogs. More recently, latanoprostene bunod was found to significantly reduce IOP and pupil diameter in normal dogs, and when compared to latanoprost these reductions were similar (Desai et al., 2022).

Latanoprostene bunod is the first FDA‐approved drug in the category of modified prostaglandin analogues and is composed of latanoprost acid linked to a NO‐donating moiety (Addis & Ellis, 2018; Desai et al., 2022; Komaromy et al., 2019). This medication was approved in the United States in 2017 for treatment of open‐angle glaucoma and ocular hypertension (Desai et al., 2022). The NO‐donating moiety, butanediol mononitrate, increases AH outflow through the conventional pathway by relaxing cells in the trabecular meshwork and Schlemm's canal (Burn et al., 2020; Desai et al., 2022). Latanoprostene bunod does not exclusively affect the conventional outflow pathway, and influences the uveoscleral pathway of AH outflow as well (Komaromy et al., 2019). The current cost of a 2.5 ml bottle is over $200 USD; however, preparation of a solitary ophthalmic NO‐donor solution could be a cost‐effective option to be used alone or in conjunction with other medications in dogs with glaucoma or post‐operative ocular hypertension.

Previous research by Nathanson has explored the efficacy of the individual topical NO donors 0.03% nitroglycerin and 0.1% hydralazine on decreasing IOP in healthy rabbit eyes (Nathanson, 1992). Nathanson's study showed that both compounded drugs were well tolerated in rabbits and reduced IOP in a significant manner, primarily by increasing drainage through the conventional pathway of AH outflow, and to a lesser extent by reducing AH production (Nathanson, 1992). Both drugs are widely available and inexpensive to produce as compounded ophthalmic formulations, so they represent a viable option for canine glaucoma; however, to the author's knowledge, no studies have been performed examining the effects of 0.03% nitroglycerin and 0.1% hydralazine on normal canine eyes.

The aim of this study was to evaluate the effect of the topical NO donors 0.03% nitroglycerin and 0.1% hydralazine on decreasing IOP in the healthy canine eye. Clinical signs of ocular irritation or discomfort were monitored, as well as heart rate to determine whether potential ocular or cardiovascular sequelae to these topical NO‐donor formulations would occur in dogs.

## 2 MATERIALS AND METHODS

Approval for this study was obtained from the Institutional Animal Care and Use Committee, and the owners of all dogs signed a statement of informed consent.

Seventy‐nine healthy, volunteered dogs were included in this study. Complete ophthalmic examinations, including slit‐lamp biomicroscopy (SL‐15 Biomicroscope, Kowa Inc, Tokyo, Japan), binocular indirect ophthalmoscopy (Heine Omega 180 Ophthalmoscope, Heine Optotechnik, Herrsching, Germany) and rebound tonometry (TonoVet^®^; Tiolat Ltd, Helsinki, Finland), were performed by a board‐certified ophthalmologist on each dog. A normal ocular examination was required prior to study inclusion. A compounding pharmacist prepared the nitroglycerin and hydralazine solutions in a sterile environment by dissolving the injectable form of the drug of interest in artificial tears (0.5% polyvinyl alcohol and 0.6% povidone; Medtech Products Inc.; Tarrytown, NY, USA) to the desired concentration. Artificial tears were used as the vehicle in all experimental groups and the control group. Once prepared, the 0.03% nitroglycerin and 0.1% hydralazine solutions were placed in separate 15 ml sterile dropper bottles (Steri‐Dropper®; The Medi‐Dose® Group, Ivyland, PA, USA) identical to the bottles used for artificial tears. A single bottle was prepared for each test drug. Bottles were stored in a refrigerator (between 36°F and 46°F) and the same bottles were used for the duration of the 8‐week study. Potency of the prepared formulations was confirmed by a third‐party testing service (Eagle Analytical, Houston, TX, USA) and repeat sample testing was performed to verify stability over 10 weeks.

The 79 dogs were divided into three groups using block randomisation: Group N (*n* = 26 dogs) was administered 0.03% nitroglycerin in one eye and vehicle‐control in the other, Group H (*n* = 26 dogs) was administered 0.1% hydralazine in one eye and vehicle‐control in the other, while Group C (*n* = 27 dogs) received vehicle‐control in both eyes (control group).

The compounding pharmacist labelled the identical sterile dropper bottles as 1A, 1B, 2A, 2B, 3A and 3B. Sub‐groups A and B were randomly made as treatment or vehicle‐control, except in the Group C where both A and B bottles were vehicle‐control. The investigators were masked as to which groups and bottles were treatment or control. Once a dog was enrolled in the study, the investigator randomly picked a number (either 1, 2 or 3) from a hat to assign a dog to a group. Subsequently, the investigator assigned a dropper bottle to each eye by picking A or B from a hat. To avoid bias in investigators’ assessment of ocular irritation and heart rate, the pharmacist who compounded the medications retained the key for the solutions’ content (i.e. whether Group N, H or C was represented by bottle number 1, 2 or 3) until the end of the experimental phase.

Rebound tonometry (TonoVet^®^; Tiolat Ltd, Helsinki, Finland) by the same skilled user was employed to measure IOP at all time points. Four acclimation readings at 5‐min intervals were collected before treatment administration, with the fourth acclimation reading serving as the baseline IOP. One baseline heart rate was measured at the time of the baseline IOP measurement. After acquiring baseline IOP for each dog, one drop (∼30 microlitres) from the randomly assigned study bottles (drug or vehicle‐control) was instilled onto each eye two times (separated by 5 min) to provide two sequential doses. IOP was subsequently measured in both eyes every 5 min for 60 min (from time point 10 to 70 min), then at 85, 100, 115, 130, 190 and 250 min after treatment. Eye drops were administered to dogs at somewhat variable times with applications initiated around 8 AM or 1 PM, and subsequent IOPs were measured over the following 4 h. All measurements were taken in one room during the 4‐h trial period, and all measurements were taken by one person throughout the study to reduce user variability.

Following treatment, heart rate was measured with a stethoscope at 15‐min intervals for 2 h, then at 190 and 250 min after treatment to assess for potential cardiovascular effects. The eyes were monitored at each IOP measurement throughout the study for conjunctival hyperaemia, blepharospasm or aqueous flare. Ocular irritation was judged subjectively as mild, moderate, and severe. A formal scoring system was not used.

### 2.1 Statistical analysis

Normality of data was assessed with the Shapiro–Wilk test. A mixed model for repeated measures was fitted to the data using the R software version 3.6.0, as previously described (Sebbag et al., 2019; Sebbag et al., 2020; Sebbag & Mochel, 2020). In the model, IOP was the response variable; the group (drug or control), time (0–250 min) and group‐by‐time interaction were treated as fixed effects; and the animal and animal‐by‐group interaction were treated as random effects, using animal as block. After the model was fit, the fixed effects were tested, and comparisons between drug versus control were made at each time point for the outcome of interest (IOP). A one‐way ANOVA was used to compare the age as well as the heart rate at each time point among Groups 1–3. Statistical analyses were performed with SigmaPlot 14.0 (Systat software, Point Richmond, CA), and *p* < 0.05 was considered significant.

## 3 RESULTS

Seventy‐nine dogs were included in this study. Group N consisted of 26 dogs (14 male neutered, 12 female spayed), with a mean ± standard deviation (range) age of 7 ± 3.7 years (2–14 years). Group H consisted of 26 dogs (9 male neutered, 16 female spayed, 1 male intact), aged 6 ± 3.4 years (1–14 years). Group C consisted of 27 dogs (14 male neutered, 12 female spayed, 1 female intact), aged 5.8 ± 3.7 years (1–13 years). Age did not differ significantly among groups (*p* = 0.444). Breeds within this study included: mixed breed (*n* = 27), Golden Retriever (*n* = 9), Chihuahua (*n* = 5), Australian Shepherd (*n* = 4), Border Collie (*n* = 3), Shih Tzu (*n* = 3), Labrador Retriever (*n* = 3), Bouvier des Flandres (*n* = 2), Boxer (*n* = 2), Greyhound (*n* = 2), Miniature Dachshund (*n* = 2), Staffordshire terrier (*n* = 2) and one each of the following breeds: West Highland White Terrier, German Wirehair Pointer, Old English Sheepdog, Basset Hound, Manchester Retriever, Catahoula, Border Terrier, Keeshond, Beagle, Miniature Schnauzer, King Charles Cavalier Spaniel, Saint Bernard, Miniature Pinscher, English Pointer, and Welsh Corgi. A post hoc sample size calculation (power 0.8, alpha 0.05) determined that the number of dogs should be 45–119 and 620–1647 to detect statistical differences in IOP within each time points for nitroglycerin and hydralazine, respectively.

**FIGURE 1:**
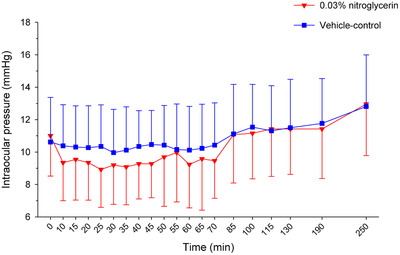
Mean ± standard deviation intraocular pressure over time in canine eyes receiving 0.03% nitroglycerin (red triangles) or contralateral eyes receiving vehicle‐control (blue squares)

**0.03% nitroglycerin (Group N) *–*** Baseline IOP did not differ significantly between eyes receiving treatment or vehicle‐control (*p* = 0.599). Eyes receiving 0.03% nitroglycerin had a significantly lower IOP over the 4‐h period when compared to vehicle‐control eyes within the treatment group (*p* < 0.0001), with a mean IOP reduction of up to 12% from 10 to 70 min post‐treatment (Figure 1). Peak reduction in IOP was noted 25 min post application and began to increase up to baseline after 70 min post‐application. However, differences in IOP between treated and vehicle‐control eyes within the treatment group were not significant at any individual time points (*p* ≥ 0.357).

**FIGURE 2:**
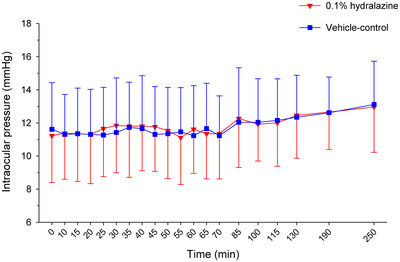
Mean ± standard deviation intraocular pressure over time in canine eyes receiving 0.1% hydralazine (red triangles) or contralateral eyes receiving vehicle‐control (blue squares)

**0.1% hydralazine (Group H) *–*** Baseline IOP did not differ between eyes receiving treatment or vehicle‐control within the treatment group (*p* = 0.106). Treatment with 0.1% hydralazine did not significantly impact IOP, whether accounting for time (*p* = 0.520) or assessing individual time points (*p* ≥ 0.133) (Figure 2).

**Ocular tolerance and heart rate *–*** No epiphora, blepharospasm, or aqueous flare was observed in any dog after administration of topical nitroglycerin or hydralazine. A mild, transient, superficial conjunctival hyperaemia was noted infrequently with hydralazine (2/26 dogs; 7%). No dogs that received nitroglycerin or vehicle‐control demonstrated conjunctival hyperaemia. No significant changes in heart rate were observed in Groups N or H compared to the control group (Group C) at the time points measured throughout the study (*p* ≥ 0.155).

## 4 DISCUSSION

A great need for effective, widely available, and cost‐effective glaucoma therapeutics exists in veterinary medicine. The aim of this study was to determine the efficacy of topical NO donors 0.03% nitroglycerin and 0.1% hydralazine on reducing IOP in healthy canine eyes as potential glaucoma or ocular hypertension therapeutics. Both compounds were well tolerated and did not significantly affect heart rate or cause appreciable signs of ocular irritation. This study showed that 0.03% nitroglycerin was effective in significantly reducing IOP when assessing the entire 4‐h trial period. However, neither NO‐donor achieved significant reductions in IOP at individual time points when compared to contralateral vehicle‐control eyes, potentially due to large variability in IOP measurements. In rabbits, Nathanson and colleagues showed that both nitroglycerin and hydralazine rapidly reduced IOP in a dose‐dependent fashion, with a peak effect at 1–2 h (Nathanson, 1992). Differences between rabbits and the present canine study could be explained by species differences in ocular anatomy and physiology (Sebbag et al., 2020). The low blink rate and the thin cornea of rabbits allow for greater drug penetration across the rabbit eye compared to dogs and humans; in fact, a study by Maurice showed that ocular drug exposure is overestimated by threefold in rabbits due to the low blink rate in this species (Maurice, 1995; Sebbag et al., 2019; Sebbag et al., 2020).

Historically, NO donors have been studied for their physiologic effects on the cardiovascular system, immune system, and central and peripheral nervous systems (Cavet et al., 2014; Cavet & Decory, 2018). In the globe and other tissues of the body, three different isoforms of NO synthase allow for constitutive and inducible production of NO, suggesting basal levels of this molecule may regulate ocular physiology in normal and diseased states (Ellis, 2011). Other studies have more specifically identified NO synthase and NO in canine globes. Samuelson described multiple isoforms of NO synthase in different regions of the normal and glaucomatous canine eye (Samuelson et al., 2001). Additionally, Källberg et al. (2007) identified elevated levels of NO present in the AH and vitreous of normal and glaucomatous canine eyes.

Nitric oxide binds to its receptor soluble guanylate cyclase resulting in the production of cyclic guanosine monophosphate (cGMP), an intracellular signalling molecule that promotes alterations in trabecular meshwork morphology and subsequent increase in AH outflow (Cavet & Decory, 2018; Ellis et al., 2009; Ellis, 2011; Nathanson, 1992). Further studies in veterinary species have shown NO induced ciliary muscle relaxation in bovine and primate species, and decreased cell volume in the porcine trabecular meshwork resulting in increased AH outflow (Dismuke et al., 2008; Gabelt et al., 2011; Wiederholt et al., 1994). No research has been published showing efficacy of NO‐donating compounds in canine patients with goniodysgenesis or those with narrow/closed angle glaucoma, as one can speculate that the drug may not alter genetically abnormal trabecular meshwork.

Given NO's many roles, it is possible to imagine that the ocular hypotensive effects of 0.03% nitroglycerin could have been mitigated by coinciding actions on other structures within the eye. To explain NO's bimodal or even hypertensive effects in the eye, Nathanson hypothesised that NO's effects on ciliary body arterial supply caused vasoconstriction and could have resulted in temporarily increased AH production when administered at higher concentrations (Nathanson, 1992). Other possibilities include relaxation of ciliary body smooth muscle in response to NO at higher concentrations, which would simultaneously decrease outflow facility and negate the hypotensive effects of these drugs (Nathanson, 1992). Further work needs to be performed to identify NO's effect on canine intraocular structures to better optimise the efficacy of these drugs.

Although 0.03% nitroglycerin and 0.1% hydralazine were ineffective in significantly reducing IOP in treated versus control eyes at individual time points in this study, nitroglycerin did have a statistically significant effect on IOP over the 4‐h trial period. It is important to acknowledge that the overall reduction of IOP and the duration of action are not clinically significant. It is conceivable that these drugs may have a more notable effect in dogs with primary open angle glaucoma or post‐operative ocular hypertension as opposed to normal dogs; however, a clinical effect is unexpected in dogs with an altered iridocorneal angle and trabecular meshwork. Potential methods of increasing efficacy of these drugs include altering drug concentrations, frequency of administration, or combining NO donors with drugs of known efficacy and different mechanisms of action. Indeed, a higher concentration of nitroglycerin or hydralazine may enhance the IOP‐lowering effect in canine eyes given the dose‐dependent effect of NO donors reported in other species (Dismuke et al., 2008; Nathanson, 1992). Additionally, recent studies exploring the use of combination therapies (e.g. latanoprostene bunod) showed that combining a drug of known efficacy with a NO‐donating moiety could provide synergistic results (Burn et al. 2020; Komaromy et al., 2019; Krauss et al., 2011).

Several ocular hypotensive agents have been proven more efficacious in glaucomatous eyes when compared to normotensive eyes (Gelatt & Mackay, 2001; Gelatt et al., 1979; Gum et al., 1991; King et al., 1991). For example, preliminary studies determined greater hypotensive effects after systemic and topical administration of multiple carbonic anhydrase inhibitors in glaucomatous Beagles than normotensive Beagles (Gelatt et al., 1979; King et al., 1991). Moreover, studies have shown the effect of topical prostaglandins on glaucomatous canine eyes were greater than their effect on normotensive eyes. Studies evaluating the efficacy of 0.03% nitroglycerin and 0.1 % hydralazine in glaucomatous canine eyes or eyes with post‐operative ocular hypertension may provide more significant results, especially if the iridocorneal angle is open rather than closed.

The present study used a large and diverse population of dogs, accounting for variability in tear film dynamics that could affect drug bioavailability following eye drop administration (Addis & Ellis, 2018; Sebbag et al., 2019). However, the study is limited by the lack of a proper acclimatisation phase, recently suggested to extend over a minimum of five training days to enhance IOP data reliability (Fentiman et al., 2019). This was not feasible for logistical reasons (volunteered dogs, not purpose‐bred dogs maintained in a colony), yet this limitation may have contributed to wide variability in IOP and impacted the ability to detect significant differences at individual time points. Additionally, this study only monitored heart rate to investigate potential systemic side effects, while future studies could consider assessing blood pressure as well given the reported vasodilating effects of NO donors. The effect of multiple IOP readings within one visit is an additional limit to this study, as repeated tonometry within one visit could result in significant reductions in IOP, though this may not be clinically appreciable (Pe'er et al., 2021). This limitation is unavoidable in studies where repeated IOP measurements are required; however, ensuring that study participants receive the same number of measurements at the same frequency can reduce variability. A final limitation of the study is that the administration of eye drops, and subsequent IOP measurements, starting at slightly different times of the day for each dog could change the reliability of the data given diurnal variation of IOP. However, such diurnal variability was accounted for by assessing the contralateral eye as control which is subject to the same diurnal variation.

## 5 CONCLUSION

In conclusion, healthy canine eyes that received 0.03% nitroglycerin had a significantly lower IOP over the 4‐h testing period when compared to vehicle‐control eyes, although the maximal IOP reduction was somewhat limited (12%). Both drugs were well tolerated with no significant signs of ocular irritation, and 0.1% hydralazine only caused a mild, transient conjunctival hyperaemia in 2/26 dogs. Nitroglycerin may be effective as an IOP‐lowering agent in canine eyes, however, further research with eyes experiencing post‐operative ocular hypertension, different forms of glaucoma, varying drug concentrations, or combination therapies may offer more significant results.

## CONFLICT OF INTEREST

The authors declare the research was conducted in the absence of any commercial or financial relationships that could be potential conflicts of interest.

## ETHICAL AND ANIMAL WELFARE STATEMENT

The authors confirm that the ethical policies of the journal, as noted on the journal's author guidelines page, have been adhered to and the appropriate ethical review committee approval has been received. The US National Research Council's guidelines for the Care and Use of Laboratory Animals were followed. Approval for this study was obtained from the Iowa State University Institutional Animal Care and Use Committee (protocol #1‐16‐8172‐K), and the owners of all dogs signed a statement of informed consent.

## AUTHOR CONTRIBUTIONS

Parker Wilcox: Investigation (equal); visualisation (lead); writing – original draft (lead); writing – review & editing (equal). Travis Strong: Conceptualisation (equal); funding acquisition (equal); investigation (equal); project administration (equal); resources (equal); supervision (equal); writing – review & editing (equal). Lionel Sebbag: Formal analysis (lead); writing – review & editing (equal). Rachel Allbaugh: Conceptualisation (equal); funding acquisition (equal); investigation (equal); project administration (equal); resources (equal); supervision (equal); writing – review & editing (equal).

### PEER REVIEW

The peer review history for this article is available at https://publons.com/publon/10.1002/vms3.945

## Data Availability

The data that support the findings of this study are available from the corresponding author upon reasonable request.
